# Thyroid nodules with Hürthle cells: the malignancy risk in relation to the FNA outcome category

**DOI:** 10.1007/s40618-019-01055-0

**Published:** 2019-05-10

**Authors:** D. Słowińska-Klencka, K. Wysocka-Konieczna, E. Woźniak-Oseła, S. Sporny, B. Popowicz, J. Sopiński, K. Kaczka, K. Kuzdak, L. Pomorski, M. Klencki

**Affiliations:** 1 Department of Morphometry of Endocrine Glands, Chair of Endocrinology, Medical University of Lodz, Pomorska Str 251, 92-213 Lodz, Poland; 2 Department of Endocrinological, General and Oncological Surgery, Chair of Endocrinology, Medical University of Lodz, Pabianicka Str 62, 91-513 Lodz, Poland; 3 Department of General and Oncological Surgery, Chair of Surgical Clinical Sciences, Medical University of Lodz, Pomorska Str 251, 92-213 Lodz, Poland

**Keywords:** Thyroid, Cancer, FNA, Oxyphilic cell, Hürthle cell

## Abstract

**Purpose:**

The aim was to find whether the presence of Hürthle cells (HC) in a smear influences the categorization of FNA results or the risk of malignancy (RoM) of particular categories of cytological diagnosis.

**Methods:**

25,220 FNA performed in a single center in years 2005–2017 were analyzed. Almost all the examined patients were exposed to moderate iodine deficiency for most of their lives. The distribution of FNA outcome categories was compared between two groups: with or without HC (HC and non-HC). The RoM was evaluated on the basis of postoperative histopathological examination (3082 patients).

**Results:**

HC were found in 7.5% of diagnostic FNA. HC nodules were classified into categories II (78.2% vs. 91.9%, *p* < 0.0000) and VI (0.4% vs. 1.2%, *p* = 0.0017) less often than non-HC nodules, but more frequently to categories III (14.4% vs. 5.8%, *p* < 0.0000), IV (11.2% vs. 0.9%, *p* < 0.0000) and V (1.5% vs. 0.8%, *p* = 0.0013). There were no significant differences in RoM between HC and non-HC nodules. The RoM in HC and non-HC nodules of particular categories of the Bethesda system was as follows: II: 1.8% vs. 0.8%, III: 9.7% vs. 3.8% when only the last FNA was considered and 10.8% vs. 6.4% when the category III in any performed FNA was considered; IV: 12.7% vs. 10.9%; V: 41.7% vs. 58.2%; and VI: 100% vs. 96.9%.

**Conclusions:**

HC nodules are classified into categories of equivocal cytological outcomes more often than nodules without HC. Nevertheless, the presence of HC in a smear does not significantly affect the RoM of FNA categories.

## Introduction

The Hürthle cells (HC) are cells with features characteristic of an oncocyte that are found in the thyroid gland. When examined by light microscopy, oncocytes correspond with epithelial cells exhibiting abundant, finely granular cytoplasm around nucleus and a variably conspicuous nucleolus [1]. The granularity of the cytoplasm is a result of the presence of innumerable, large, often vacuolated and dilated mitochondria [2]. Mitochondrial proteins exhibit avidity to bind acidic dyes like eosin. Due to this the oncocytes have also been termed oxyphils.

The HC found in the thyroid may be scattered or they may constitute foci. The cells are present both in non-neoplastic lesions: nodular goiter (especially in elderly), Hashimoto disease, Graves’ disease, lesions induced by radiotherapy or systemic chemotherapy and in thyroid neoplasms [1, 2]. Apart form Hürthle cell adenomas (HTA) or Hürthle cell carcinomas (HTC), which are neoplasms composed of at least 75% HC, these cells may be present in follicular thyroid adenomas (FTA), follicular thyroid carcinomas (FTC) and papillary thyroid carcinomas (PTC). In the case of the latter, it may be difficult to differentiate between HC and PTC cells, which usually show oncocyte-like oxyphilic cytoplasm [1, 2]. Until recently, Hürthle cell neoplasm was considered a subtype of follicular neoplasm. The recent WHO classification defines them as a separate entity because of the clinical, pathological, and molecular profiles that differ from the ones of follicular neoplasm [3].

The preoperative diagnostics of the HC lesions with the use of fine-needle aspiration biopsy (FNA) is difficult. The cytological criteria for Hürthle cell neoplasm included the absence or scarcity of colloid and the absence of lymphoplasmacytic infiltration, along with the presence of hypercellularity and the exclusivity of oncocytes, small cell dysplasia, large cell dysplasia, dyscohesive single cell pattern, crowded syncytial arrangements and, in some cases, transgressing vessels. Unfortunately, there are no unequivocal criteria for distinguishing HTC from HTA [4]. The FNA outcome in these cases is equivocal and it is usually classified into the category IV: suspicion of follicular neoplasm (SFN), suspicion of Hürthle cell tumor (SHCT) or the category III: follicular lesion of undetermined significance (FLUS)/atypia of undetermined significance (AUS) of the Bethesda System for Reporting Thyroid Cytopathology (BSRTC) [5]. In some centers, in the case of the category III, the subcategory of Hürthle cell FLUS (HC-FLUS) is used to underline the predomination of HC in the smears [6–14]. However, smears containing HC may be classified into each of the six Bethesda System categories depending on the microscopic image, with a higher prevalence of suspicious/malignant cytology in the case of patients with Hashimoto disease [15, 16].

The analysis of the malignancy risk related to categories III and IV and its dependence on the presence of HC in smears was the subject of some studies that did not reach unequivocal conclusions. The category IV of FNA outcomes is an indication for surgical treatment. Among surgically excised nodules reported as SFN/SHCT the malignancy rate is 10–40% [17]. Some researchers indicate that the risk of malignancy is higher for SHCT than SFN [18, 19]. There are also opposing reports [20] that show similar malignancy risk [21, 22]. In the case of the category III, it is more difficult to obtain reliable data. This category is not an indication for surgical treatment but for performing control FNA and molecular tests if available, although the efficacy of those tests in the case of nodules with predominant HC is less known [17, 23, 24]. Consequently, the data on the incidence of malignancy among these lesions are mainly based on the clinical follow-up instead of histopathological examination. Additionally, the exact way of classification of smears into the category III is highly variable, which makes the estimated risk of malignancy vary from several up to nearly 70% [6, 9, 22, 25, 26].

Another factor that makes the comparison of obtained results difficult is the iodine supply in the examined population. Iodine modifies the relative frequency of non-neoplastic and neoplastic thyroid lesions, as well as the relative incidence of PTC and FTC [27]. Consequently, the iodine supply influences the numbers of nodules classified into the categories III and IV as well as the risk of malignancy in these groups.

The aim of the study was to find whether the presence of HC in a smear influences the categorization of FNA results and the risk of malignancy in the thyroid nodules of particular cytological categories.

## Materials and methods

### Examined patients

The analysis included the results of 25,220 FNA performed in a single center in years 2005–2017. The biopsies performed in years 2005–2009 (first period) and in years 2010–2017 (second period) were analyzed separately. In the first period, FNA was performed in 11,598 patients and their results were classified into the 6 categories described below. In the second period, FNA was performed in 13,622 patients and their results were formulated according to the BSRTC. The mean patient age in the first period was 2 years lower than in the second one (56.5 ± 13.2 vs. 58.6 ± 13.3 years, respectively; *p* < 0.0000). The percentage of males in the first period was about 1% lower than in the second one—respectively: 10.0% (1157) vs. 10.9% (1503), *p* < 0.0147. Almost all the examined patients were exposed to moderate iodine deficiency for most of their lives. According to the criteria of the International Council for Control of Iodine Deficiency Disorders, in the 1990s, our country was classified as a moderate iodine-deficient area. Mandatory iodization of household salt was introduced in 1997. The efficacy of that prophylaxis in lowering the prevalence of goiter among school-aged children below < 5% was confirmed as early as 2005 [28]. Consequently, over 98% of patients examined in the first period were exposed to moderate iodine deficiency for at least half of their life (the period of sufficient iodine supply in that group was 12 years maximum, and patients under 24 constituted only 1.3% of the examined group). Ninety percent of patients examined in the second period were in the similar situation (the period of sufficient iodine supply in that group was 20 years maximum and patients under 40 constituted 10.0% of the examined group).

The FNAs were carried out in patients referred by endocrinologists from outpatient clinics. All the biopsies were US-guided. FNAs were performed on nodules with a diameter of at least 5 mm (usually over 1 cm), which were palpable or had at least one malignancy risk factor (ultrasonographic or clinical). Two aspirations of a nodule were usually performed. Smears were fixed with 95% ethanol solution and stained with haematoxylin and eosin. Surgical thyroidectomy specimens were processed by standard procedures.

### Description of the classification of cytological outcomes

In years 2010–2017, the Bethesda classification with six categories was used. In this classification, the category I includes non-diagnostic biopsies (ND), the category II—benign lesions (BL), the category III—FLUS and HC-FLUS, the category IV—SFN and SHCT, the category V—suspicious for malignancy (SM), and the category VI—malignant neoplasm (MN). Specimens showing a prominent monotonous population of thyroid follicular cells (tfc) arranged in three-dimensional groups and microfollicles with nuclear overlapping and crowding in background of scant or no colloid or containing single cell population of oncocytic cells ( > 75% of cells) with prominent nucleoli arranged in sheets and cohesive groups were classified as SFN or SHCT. The category IV did not include lesions with nuclear features of PTC. The diagnosis of FLUS or HC-FLUS was made when the specimen showed features from the borders of the categories II and IV. Very rare cases with the presence of local features suggestive of PTC (nuclear grooves, enlarged nuclei with pale chromatin and alterations in nuclear contour and shape) in an aspirate that was otherwise benign in microscopic appearance or specimens with limited cellularity but with nuclear atypia were classified into the category III of the BSRTC.

In years 2005–2009, a similar classification with six categories was used. The categories I, II, IV, V and VI were formulated analogically to the BSRTC categories. Instead of SFN and SHCT, the terms “follicular neoplasm” (FN) and “Hürthle cell tumor” (HCT) were used. In some cases, the cytopathologist tried to determine the benign character of the follicular lesion more precisely by formulating the result as “follicular neoplasm probably benign” (FNpB) and for lesions with predomination of HC—HCT-pB. Diagnoses of FNpB and HCT-pB were included in the category III of that classification. Smears with monomorphic tfc but with cytological picture not allowing the formulation of a diagnostic conclusion were also assigned to this category, for the purpose of a comparative analysis with the results of postoperative histopathological examinations. For the same reason, the smears with single features characteristic of the thyroid cancer, especially features of PTC, were classified into the category V.

In both evaluated periods, when several nodules were examined, the FNAB outcome was classified according to the one related to the highest risk of malignancy.

### Types of analysis

The distributions of FNA results among particular categories of cytological diagnoses in relation to the presence of HC in the smears (HC and non-HC groups) were compared between the examined periods—2005–2009 and 2010–2017. This analysis included the results of the first FNA only, without considering control examinations. Non-diagnostic smears were excluded from the analysis.

The cytological results of FNA performed in patients who subsequently underwent the thyroid surgery were then analyzed. The category distribution of FNA results was analyzed for surgically resected HC and non-HC nodules. The risk of malignancy (RoM) was assessed as a rate of cancers found with postoperative histopathological examination in the nodules. The risk was calculated in relation to the cytological diagnostic category of FNA as well as independently of the category of FNA (total RoM—tRoM). The types of cancers identified in HC and non-HC nodules were compared. Those analyses concerned the results of the last FNA performed before the surgery. The assessment of RoM for the category III was additionally done in a way that included all the resected nodules for which any FNA outcome (first or control one) was regarded as FLUS or HC-FLUS. The analysis excluded patients with incidental cancers revealed in postoperative histopathological examination.

The statistical analysis was performed with the Statistica, version 10 statistical software. The comparison of frequency distributions was performed with the *χ*^2^ test (with suitable modifications according to the number of analyzed cases) and the Kruskal–Wallis test was used for the comparison of continuous variables between groups. The value of 0.05 was assumed as the level of significance. The study protocol had been approved by the local Bioethics Committee. All the patients gave their informed consent to perform FNA.

## Results

The frequency of finding HC in the aspirates was similar in both of the analyzed periods: 7.4% (867 patients) in the first one, 7.5% (1018 patients) in the second one, *p* = 0.9947. In the whole examined timespan (years 2005–2017), HC nodules were classified into categories II and VI less often than non-HC nodules, but more frequently into the categories IV and V. In the case of categories II and IV, the differences were significant in each of the two examined periods, in the case of the category V—only in the first period, and category VI—only in the second period (Table 1).

**Table 1:** Distribution of diagnostic FNA outcomes among particular categories of cytological diagnoses in both of the analyzed periods for HC and non-HC nodules^*,**^

Category of FNA	No/% of patients with diagnostic cytology		*p*
	HC smears	Non-HC smears	
Before Bethesda classification—2005–2009			
II	729/84.1	9920/92.4	< 0.0000
III	16/1.8	477/4.4	0.0003
IV	104/12.0	135/1.3	< 0.0000
V	17/2.0	102/1.0	0.0045
VI	1/0.1	97/0.9	0.0672
Total	867/100	10,731/100	
After Bethesda classification—2010–2017			
II	745/73.2	11,535/91.5	< 0.0000
III	147/14.4	731/5.8	< 0.0000
IV	108/10.6	76/0.6	< 0.0000
V	12/1.2	90/0.7	0.0980
VI	6/0.6	172/1.4	0.0362
Total	1018/100	12,604/100	
Both periods overall—2005–2017			
II	1474/78.2	21,455/91.9	< 0.0000
III	163/8.6	1208/5.2	< 0.0000
IV	212/11.2	211/0.9	< 0.0000
V	29/1.5	192/0.8	0.0013
VI	7/0.4	269/1.2	0.0017
Total	1885/100	23,335/100	

*Only first FNA in a patient was considered, without taking control examinations into account

**When several nodules were examined, the FNA outcome was classified according to the one related to the highest risk of malignancy

The introduction of the Bethesda system decreased the incidence of the category II for HC and non-HC nodules (HC: 84.1% vs. 73.2%, *p* < 0.0000; non-HC: 92.4% vs. 91.5%, *p* = 0.0098) and increased the incidence of the category III (HC: 1.8% vs. 14.4%, non-HC: 4.4% vs. 5.8%, *p* < 0.0001 both). The smears classified into the category II showed features of chronic thyroiditis (CT) more frequently in the HC group than in the non-HC group: the first period—51.3% (374 patients) vs. 8.4% (830), *p* < 0.0000; the second period—63.5% (473 patients) vs. 5.5% (631), *p* < 0.0000, respectively. The increase of CT features in the HC group was statistically significant (*p* < 0.0000). The new category III amounted to 6.4% of all FNA results and was more common among HC nodules (14.4%) than non-HC ones (5.8%), unlike the previous category III (Table 1). The smears with nuclear atypia constituted 2.7% (4) of HC nodules classified into the new category III and 1.9% (14) of non-HC nodules. Non-HC nodules were classified into categories IV and V less frequently (IV: 1.3% vs. 0.6%, *p* < 0.0000, V: 1.0% vs. 0.7%, *p* = 0.0463), but more frequently into the category VI (0.9% vs. 1.4%, *p* = 0.0010) after the introduction of the Bethesda system. The group of HC nodules showed similar but not statistically significant tendencies.

The patients with HC nodules were on average a year older than the patients with non-HC nodules (58.8 ± 13.4 vs. 57.6 ± 13.3 years, *p* < 0.0000). The percentage of males in the HC group was lower than that in the non-HC group: 5.9% (112) vs. 10.9% (2552), *p* < 0.0000. The differences in the percentage of males were significant only in the category II (HC: 4.7% vs. non-HC: 10.9%, *p* < 0.0000).

Table 2 shows the comparison of malignancies found with histopathological examination in HC and non-HC nodules. The analysis of examined timespan taken as a whole (years 2005–2017) and as two separate periods did not show any difference between HC and non-HC nodules in the total RoM. The incidence of the category II decreased in the second period and the total RoM in the second period was higher than in the first one in both types of nodules (HC: 14.6% vs. 7.9%, *p* = 0.0487; non-HC: 11.2% vs. 6.6%, *p* = 0.0001). That decrease was more striking in HC than non-HC group (HC: 56.4% vs. 29.7%, *p* < 0.0000; non-HC: 82.4% vs. 71.7%, *p* < 0.0000). The incidence of the category VI for non-HC nodules increased in the second period (5.2% vs. 7.7%, *p* = 0.0127); HC nodules showed a similar tendency (1.8% vs. 4.3%, *p* = 0.3009).

**Table 2:** Comparison of the rate of malignancy determined by postoperative histopathological examination in HC and non-HC nodules in relation to the category of FNA result^*,**^

Category of FNA results	Results of histopathological examinations—No/% of patients				*p*
	HC smears		Non-HC smears		
	Benign	Malignant	Benign	Malignant	
Last FNA before Bethesda classification—2005–2009					
II	89/95.6	4/4.3	875/99.4	5/0.6	0.0026
III	4/100.0	–	19/100.0	–	–
IV	53/91.4	5/8.6	67/90.5	7/9.5	0.8679
V	6/85.7	1/14.3	33/84.6	6/15.4	0.6193
VI	–	3/100	3/5.4	53/94.6	0.3486
Total	152/92.1	13/7.9	997/93.4	71/6.6	0.5592
Last FNA after Bethesda classification—2010–2017					
II	54/98.2	1/1.8	1184/99.2	9/0.8	0.9269
III	28/90.3	3/9.7	204/96.2	8/3.8	0.3104
IV	69/87.3	10/12.7	57/89.1	7/10.9	0.7519
V	7/58.3	5/41.7	28/41.8	39/58.2	0.2881
VI	–	8/100	4/3.1	124/96.9	0.5680
Total	158/85.4	27/14.6	1477/88.8	187/11.2	0.1758

*When several FNA were performed in a patient, the outcome of the last one was considered

**Statistical analysis did not include incidental cancers

The RoM in HC and non-HC nodules within particular FNA result categories treated as an indication for surgical treatment (IV, V and VI) was similar (Table 2). The RoM in category V nodules was higher in the second period than in the first one—that difference was significant for non-HC nodules: 58.2% vs. 15.4% (*p* < 0.0000) and insignificant for HC nodules: 41.7% vs. 14.3% (*p* = 0.1633). In the case of the category II, the RoM was higher in HC than non-HC nodules in the first period (4.3% vs. 0.6%, *p* = 0.0026) but similar in the second period (1.8% vs. 0.8%, *p* = 0.9269). In the case of the category III, no HC nodule nor non-HC one was found to be malignant in the first period. After the modification of the category III according to the BSRTC, the incidence of the category III in operated patients increased (HC: 2.4% vs. 16.8%, *p* < 0.0000; non-HC: 1.8% vs. 12.7%, *p* < 0.0000). When the results of the last FNA before the operation were considered, the RoM in the category III of the Bethesda system was 9.7% for HC nodules and 3.8% for non-HC nodules (*p* = 0.3104). When the results of all FNA (first and control) were analyzed, it increased to 10.8% (4 of 37) for HC nodules and to 6.4% (15 of 235) for non-HC nodules (*p* = 0.5253). In the category III, there were eight non-HC nodules with cytological features of nuclear atypia. Cancer was diagnosed in one of them (12.5%. vs. 3.5% in other non-HC nodules of the category III, *p* = 0.7079). None of the four patients in the HC group with features of nuclear atypia in the smears were treated surgically.

Malignant HC nodules corresponded to HTC more often than malignant non-HC nodules, but to PTC—less often (Table 3). Nevertheless, PTC was the most common cancer in both groups. The percentage of PTC among cancers did not significantly differ between HC and non-HC nodules in each of the analyzed categories of FNA result. The percentages were the closest in the case of the category IV (HC: 40.0%, non-HC: 35.7%) and V (HC: 83.3%, non-HC: 84.4%); in the other categories they were as follows: category II—HC: 20.0%, non-HC: 42.9%; category III—HC: 33.3%, non-HC: 75.0%, category VI—HC: 63.6%, non-HC: 79.1%. Most HTC were diagnosed in HC nodules classified to the category IV (5), and the other HTC were found in nodules of categories V (1), III (1), II (1) and in one non-HC nodule of the category VI.

**Table 3:** Types of cancers found histopathologically in HC and non-HC nodules

Types of malignant neoplasms	No/% of patients with malignant neoplasm		
	HC smears	Non-HC smears	*p*
PTC	21/52.5	195/75.6	0.0024
FTC	6/15.0	18/7.0	0.0827
HTC	8/20.0	1/0.4	< 0.0000
MTC	2/5.0	15/5.8	0.8730
ATC	1/2.5	8/3.1	0.7719
Lymphoma	–	8/3.1	0.5463
Epithelioid angiosarcoma	–	3/1.2	0.8684
Adenoid cystic carcinoma	–	1/0.4	0.2825
Secondary tumors	2/5.0	9/3.5	0.9381
Total	40/100	258/100	

There was no difference in the percentage of males in the operated patients between the HC and non-HC groups: 10.6% (37) vs. 12.8% (350), *p* = 0.2338. The percentage of males with cancers and with benign lesions in the HC group was 15.0% (6) vs. 10.0% (31), respectively (*p* = 0.3331), and in the non-HC group—16.3% (42) vs. 12.4% (308), *p* = 0.0799. In both of the HC and non-HC groups, the mean ages of patients with cancers and of the patients with benign lesions were similar—HC: 54.4 ± 14.3 vs. 55.5 ± 13.2 years (*p* = 0.5843); non-HC: 53.2 ± 12.1 vs. 52.7 ± 14.5 (*p* = 0.3358).

## Discussion

Our results confirm that the preoperative cytological diagnostics of Hürthle cell nodules is not easy. The smears in which HC are present are classified into the unequivocal categories II and VI less often but more frequently into the equivocal categories IV and V. That difference is especially pronounced in the case of the category IV. That category was 10 times more common among HC nodules than non-HC ones in both of the analyzed periods. The HC nodules were also classified into the new equivocal category III of FNA outcomes introduced by the Bethesda system more than twice as often as the non-HC nodules. The new category is meant to include no more than 7% of FNA results and this threshold was achieved in our center. However, the percentage of category III results in the group of HC nodules is nearly two times higher (14.4%). Similar data indicating a more frequent classification of smears with HC into equivocal categories were shown by Yazgan et al. [29].

Our results also indicate that the introduction of the new category III decreased the numbers of FNA results classified into categories II, IV and V for both types of nodules (HC and non-HC). That is because the category III includes specimens in which the cytomorphological findings are not representative of a BL, yet the degree of cellular, nuclear and/or architectural atypia is not sufficient to render a diagnosis of SFN/SHCT or SM. Changes in the number of FNA results assigned to the category II were more pronounced in the case of HC nodules (over 10%) than non-HC nodules ( < 1%). Prior to the introduction of the Bethesda system, HC nodules diagnosed as benign eventually proved to be cancers more often than cytologically benign non-HC nodules. Currently, the classification of a HC nodule into the category II has a higher negative predictive value that is close to the one found for non-HC nodules and exceeds 98%. In relation to the diagnostic value of the category V, the consequences of the introduction of the category III were also positive: its positive predictive value increased. A similar impact of the new category III on the frequencies of categories II, IV and V as well as the risk of malignancy related to the category V was reported by Kiernan et al. [26]. In our population, the effect of modified categorization of FNA results could be increased by the effective introduction of iodine prophylaxis. An increased iodine supply leads to a decrease in the incidence of non-neoplastic and neoplastic follicular lesions and an increase of the relative incidence of PTC among thyroid cancers [27]. Accordingly, we observed an increase in the number of category VI FNA outcomes. This effect may be partly attributed to the improved assessment of ultrasonographic malignancy risk features that is especially useful in the diagnostics of PTC [30].

The increased iodine supply in our population also resulted in changes in the distribution of FNA outcome categories in the patients referred for the surgical treatment. This group of patients was dominated by those with a cytological diagnosis of BL which is characteristic of populations exposed to iodine deficiency. However, their percentage decreased between the first and second periods. That decrease was more conspicuous in the HC group (about 27%) than in the non-HC one (about 11%) because of a parallel increase in the percentage of cytological diagnoses of CT in patients with HC nodules. That increase is typical of regions with increasing iodine supply [27]. CT is rarely accompanied by a large or suspicious goiter that could suggest necessity of the surgical treatment. Different incidences of CT in both sexes, with a higher morbidity among women, were the cause of differences in the distribution of males and females in HCs and non-HCs groups. The percentage of male patients in the HC group was nearly two times lower than in the non-HC group.

In our material, the total RoM as verified by a histopathological examination did not differ significantly between HC and non-HC nodules but in both periods it was higher for HC nodules. Data published on the malignancy risk of HC nodules are unclear; some researchers indicate the existence of the increased risk related to such nodules [18, 31], while other do not confirm this suggestion [21, 22]. It should be underlined that the precise assessment of this risk is difficult. Unfortunately, many published reports lack the data on the RoM in HC nodules in relation to the FNA outcome category. The majority of the papers only concern the category IV, but even so they usually give no information on the intensity of the oncocytic changes. Some centers adopt a 50% threshold of HC percentage, whereas others require a far more stringent 90%. A commonly used definition for the diagnosis of SHCT assumes that a smear should contain at least 75% of HC with little or no background colloid; the nuclear features of PTC must be absent [2]. This definition was also used at our center. But the 2017 BSRTC includes a modification to the diagnostic criteria for this category. Currently, follicular-patterned cases with mild nuclear changes (increased nuclear size, nuclear contour irregularity, and/or chromatin clearing) can also be classified into the category IV as long as true papillae and intranuclear pseudoinclusions are absent [17]. This approach is similar to the Italian consensus on cytological subclassification of thyroid indeterminate nodules in which the category TIR 3B corresponds to the category IV according to the BSRTC, but it also includes the cases with “mild/focal nuclear atypia” [32, 33]. In our material, the Bethesda system introduced prior to that modification was used and no difference was found in the RoM in HC and non-HC nodules among particular categories of FNA outcome. Similar observations were reported by Yazgan et al. [29] in relation to categories other than the category IV. In the category IV, they found higher RoM in nodules with HC present. The authors attributed that finding mostly to a misinterpretation of PTC cells as HC. Nearly, three-fourth cancers in the Hürthle cell group were diagnosed as PTC in their study. Arduc et al. [34] reported an even higher (86.6%) percentage of PTC among cancers in the nodules diagnosed cytologically as Hürthle cell lesion/neoplasms. In our material, PTC constituted 52% of the cancers in the HC group, and the percentage of PTC among cancers in HC and non-HC nodules classified into the category IV was similar and did not exceed 40.0%. A similar RoM of category IV nodules with or without HC was reported by Harvey et al. [35] (27% vs. 21% respectively), Deandra et al. [36] (22.8% vs. 25.0%), Sorrenti et al. [37] (18.6% vs. 17.3%), Sclabas et al. [38] (20.0% vs. 15.0%) and Castro et al. [39] (14% vs. 16%). A higher but statistically insignificant difference was found by Raparia et al. [31] (41% vs. 24%). Sangalli et al. [19] reported a significant difference (32.1% vs. 15.5%, respectively).

The RoM of HC and non-HC nodules classified into the category IV did not exceed 15% in our material and was lower than the usually reported values [18, 21, 22, 25, 26, 31, 34, 40]. This may be related to the fact that 90% of the patients diagnosed at our center were exposed to a long-term iodine deficiency. Non-neoplastic follicular lesions, especially hyperplastic nodules, are predominant thyroid lesions in such a population. Similarly, a low rate of malignancy for the cytological diagnosis of Hürthle cell neoplasm (6.0%) was reported by Rossi et al. [41] for an Italian population. Interestingly, Ito et al. [42], Lee et al. [40] and Castro et al. [39] reported only 15% RoM for the category IV in populations with a very high or high iodine supply (Japanese, Korean and American, respectively). It is probable that the authors avoided assigning smears with cells presenting oncocyte-like cytoplasm and features of PTC into the category IV, which is suggested by a low percentage of PTC among histopathologically diagnosed cancers.

In our material, the new category III had a higher RoM in the HC nodules than in the non-HC nodules (9.7% vs. 3.8% when only the last FNA was considered; 10.8% vs. 6.4% when the category III in any performed FNA was regarded). Those differences were not statistically significant but they would probably become significant if the number of examined patients were increased. A similar risk of malignancy associated with the category III with a domination of HC was reported by McKee et al. [22], but the authors did not compare it with the non-HC group. It should be stressed that the clinical interpretation of the category III varies between particular centers because of very different RoM values ranging from several up to nearly 70%. It is the consequence of an imprecise definition of this category, which leads to poor interobserver reproducibility [14]. In the centers, where the category III is dominated by smears with nuclear atypia, including features typical of PTC, the RoM is higher [6, 43] than in populations similar to ours where the category III usually includes nodules with disturbed cellular architecture from the border between categories II and IV. But even here the rare cases with nuclear atypia are characterized by a higher RoM. It seems reasonable to identify at least three subgroups within the category III to perform a deeper analysis: with nuclear atypia, with architectural atypia and with HC predominance [17]. But even such a detailed division does not make reported results consistent. Some studies showed that the RoM in the subgroup with the predominance of HC and in the nodules with architectural atypia is similar and lower than the RoM observed in the nodules with nuclear atypia [6–9]. Another study indicated that the RoM in the subgroup with HC predominance is similar to that observed in the subgroup with nuclear atypia and higher than the one in nodules with architectural atypia [10]. Some other studies showed that the RoM of “atypia, rule out Hürthle cell neoplasm”/“AUS cannot exclude Hürthle cell neoplasm” was lower than the one in the two other subgroups of the category III [11–14].

Those differences may also be attributed to the way of assessing RoM for the category III. In the case of other categories, the RoM is usually related to the result of the last FNA performed before the surgery (it is frequently the only FNA). But in the case of the category III, this method leads to omissions of cases in which subsequent FNA changes classification of the outcome from the category III to another category, usually a more suspicious one. That is why in our study, the analysis of RoM included all (first or control) FNA classified as FLUS. It should be kept in mind that the real RoM of the categories that are not an indication for the thyroid surgery is lower than the incidence of cancer in the postoperative histopathological examination. FLUS nodules are usually resected only if the outcome of control FNA is more suspicious or there are other malignancy risk features. The analysis of diagnostic efficacy of control FNA was not a part of the present study but our previous reports show that in the case of FLUS nodules control FNA examinations make the diagnosis more precise in over 70% of all cases and are effective in revealing cancers [44].

In summary, nodules with HC present in smears constitute less than 10% of all nodules examined with FNA in patients exposed to iodine deficiency. Such nodules are classified into categories of equivocal cytological outcomes more often than nodules without HC. Nevertheless, the presence of HC in a smear does not significantly affect the incidence of cancers revealed in the operated patients. Specifically, the risk of malignancy assessed in this way is similar for both HC and non-HC nodules of the categories II, IV, V and VI in the Bethesda system. In the case of the category III, there are some differences that suggest the risk of malignancy in the HC nodules may be higher than in the non-HC nodules. Further analyses based on larger groups of patients with nodules belonging to this category are needed to clarify this issue.

## Data Availability

The datasets generated during and/or analyzed during the current study are available from the corresponding author on reasonable request.
